# Prospective longitudinal quality of life and survival outcomes in patients with advanced infiltrative hepatocellular carcinoma and portal vein thrombosis treated with Yttrium-90 radioembolization

**DOI:** 10.1186/s12885-017-3921-1

**Published:** 2018-01-12

**Authors:** Minzhi Xing, Nima Kokabi, Juan C. Camacho, Hyun S. Kim

**Affiliations:** 1 Interventional Radiology, Department of Radiology and Biomedical Imaging, Yale University School of Medicine, New Haven, CT USA; 2 Interventional Radiology, Department of Radiology, the Medical University of South Caroline, Charleston, SC USA; 3 Yale Cancer Center, Yale University School of Medicine, 330 Cedar Street, TE 2-224, New Haven, CT 06510 USA

**Keywords:** Hepatocellular carcinoma, Quality of life, Yttrium-90 radioembolization, Locoregional therapies

## Abstract

**Background:**

To determine the effect of Yttrium-90 (Y90) radioembolization on health-related quality of life (HRQOL) and its effect on overall survival advanced, unresectable infiltrative hepatocellular carcinoma (HCC) patients with concurrent portal vein thrombosis (PVT).

**Methods:**

Consecutive patients with unresectable infiltrative HCC and PVT were recruited. The Short-Form 36 (SF-36) questionnaire was used to assess HRQOL for consecutive patients treated with glass-based Y90 based on a prospective phase II trial. MR imaging was used to determine tumor progression every 3 months post-treatment. Overall survival (OS) from treatment and time to progression (TTP) was analyzed using Kaplan-Meier estimation and log-rank test.

**Results:**

Thirty patients were treated and followed for 17.4 months; physical and mental component summary scores (PCS & MCS) remained unchanged at one, three, and six months. While no difference was observed in baseline SF-36 scores for patients with prolonged TTP (≥4 months) and OS (≥ 6 months), corresponding 1-month PCS were significantly higher than those with TTP < 4 months and OS < 6 months. At 1 month, patients with normalized Physical Function (PF), Role Physical (RP) and PCS within 2 standard deviations (SD) of US normalized baseline scores had a significantly prolonged median OS (15.7 vs. 3.7 months; *p* < 0.001) and TTP (12.4 vs. 1.8 mo; *p* < 0.001) compared those with physical component scores greater than 2SD below normalized US population values.

**Conclusion:**

Y90 radioembolization for HCC demonstrated long-term preservation of HRQOL. Lower baseline HRQOL scores were predictive of poorer OS. Early (1 month post-treatment) significant decreases in PCS were independent predictors of poorer OS and TTP.

**Trial registration:**

ClinicalTrials.gov identifier NCT01556282, registered March 16, 2012.

## Background

Worldwide, hepatocellular carcinoma (HCC) ranks as the sixth most prevalent cancer and causes the third most cancer-related deaths [1–3]. At diagnosis, approximately 10 to 40% of HCC patients have portal vein thrombosis concurrently [4, 5]. These patients often have very poor prognosis, and are not eligible for curative surgery via resection or transplantation. Without treatment, the reported median overall survival (OS) for Barcelona Clinic Liver Cancer (BCLC) Stage C patients range from 2 months to 4.8 months, as compared to 10 to 24 months in HCC patients without PVT [5–9].

Due to the risk of hepatic artery infarction, embolic intra-arterial therapies such as transarterial chemoembolization (TACE) have previously been deemed as contraindications in HCC with PVT [10, 11]. However, more recent studies establishing the safety and efficacy of conventional TACE (cTACE) in prolonging survival for HCC patients with PVT have been conducted [12, 13]. Nevertheless, increased survival benefits have not been specifically established in infiltrative HCC patients [14]. In long-term studies, selective intra-arterial yttrium-90 (^90^Y) radioembolization has demonstrated equal or better overall survival (OS) and time to progression (TTP) vs. cTACE [15], with comparable safety and efficacy in patients with infiltrative HCC with PVT [16]. Given the lowered risk of hepatic ischemia and decreased embolic effect with ^90^Y radioembolization compared to cTACE, some authors have noted that it may be a preferred therapeutic option for patients with concurrent PVT [10, 12, 13].

In palliative oncology, health-related quality of life (HRQOL) has become an increasingly relevant measure of patient prognosis. In recent years, a progressively higher number of investigators have suggested using HRQOL as an independent prognostic factor for response to treatment and progression of disease in patients with advanced HCC [17, 18]. As a widely-used tool in the evaluation of HRQOL, the Short-Form 36 (SF-36) contains 36 items/8 domains, including physical and social well-being, vitality, and pain [19]. In HCC patients, significant improvements in mental health at four months post-therapy [20] have been reported using the SF-36 for patients treated with cTACE, while significant increases in HRQOL with ^90^Y radioembolization have been observed at one month following therapy [21]. However, to date no studies have demonstrated both survival outcomes of ^90^Y therapy and its effect on HRQOL in unresectable, infiltrative HCC with PVT in particular. Our purpose was to investigate the effect of ^90^Y radioembolization on HRQOL in patients with infiltrative, unresectable HCC with PVT, and its effect on overall survival (OS) and time to progression (TTP). We hypothesized that patients with higher HRQOL scores prior to therapy would demonstrate more favorable survival outcomes, with maintenance of HRQOL post-therapy.

## Methods

### Study design and patient selection

In an Institutional Review Board (IRB) approved correlative study based on a prospective phase II trial (trial registration at ClinicalTrials.gov; identifier NCT01556282, registered March 16, 2012), consecutive patients with infiltrative HCC with PVT were enrolled for possible therapy with glass-based ^90^Y radioembolization. Diagnosis of HCC was made according to American Association for Study of the Liver Diseases (AASLD) using dynamic contrast-enhanced magnetic resonance imaging (MRI). All patients in this study had PVT due to tumor invasion. As previously described, infiltrative HCC was defined as a geographic region with high T2 signal, arterial enhancement and early washout on T1 gadolinium-enhanced images [22]. A multidisciplinary liver cancer tumor board evaluated every patient for his/her suitably to undergo ^90^Y radioembolization.

### Pre-treatment evaluation, planning, inclusion/exclusion criteria, and ^90^Y therapy

As previously described by our group, patients who were ≥18 years with Eastern Cooperative Oncology Group (ECOG) performance score (PS) ≤2 and life expectancy of at least 3 months were included. Additionally, those with tumor burden <75% of entire liver, and adequate liver reserve based on liver function tests (serum bilirubin <2 mg/dl, serum albumin >2.5 g/dl, and/or AST or ALT <5 times the upper limit of normal) were included. Following lung shunt study, those with estimated lung dose >30Gy, lung shunt fraction (LSF) >20%, observable uncorrectable GI flow on diagnostic angiogram were excluded from this study as previously described by the authors [23]. In general, 120 Gy dosimetry to the treated lobe of the liver was planned based on Therasphere® (Biocampatibles, London, UK) published guidelines [12, 23].

### Clinical follow-up and health-related quality of life assessment

Patients were followed up in the Interventional Oncology Clinic at one week post-therapy, and subsequently at one month, three months, and every three months thereafter until death. The SF-36 Health Survey Form (version 1) assessment tool was used to measure HRQOL scores at the initial pre-treatment visit and at each subsequent post-therapy 1-, 3- and 6-month follow-up visit, with a conversion of raw scores to a 0–100 scale [19]. In line with the SF-36 Health Survey Manual and Interpretation Guide [17], the survey was self-administered by patients and scored independently.

### Statistical analysis

Overall mean scores were compared at each time point. HRQOL scores of each domain pre- and post-therapy were compared using paired t-tests. The chi-squared (χ^2^) test was used to compare categorical variables. Using the Kaplan-Meier method overall survival (OS) times were calculated from initial ^90^Y procedure. SAS version 9.3 (SAS Institute Inc., Cary, NC, USA) software was used for all computations.

## Results

### Overall baseline characteristics

Between 2011 and 2013, thirty patients with advanced, infiltrative HCC with PVT received glass-based ^90^Y radioembolization. All patients in this study had PVT due to tumor invasion. Detailed patient demographics, tumor morphology, and characteristics of portal vein thrombosis at initial presentation are shown in Table 1. Median age at baseline was 62 years (range 35–92), with the majority being male (*n* = 23; 77%) and of Caucasian ethnicity (*n* = 24; 80%). At baseline, 20 patients (67%) had documented Child Pugh A disease, and the majority had an Eastern Co-operative Oncology Group (ECOG) performance score of 1 (*n* = 17; 57%). All 30 patients demonstrated PVT on MR imaging and were thus classified as BCLC Stage C, with most patients demonstrating occlusive PVT (*n* = 23; 77%) occurring in a branch of the portal vein (*n* = 24, 80%). Twenty-one patients (70%) had evidence of hepatic cirrhosis and/or portal hypertension on baseline MR imaging. Twenty-two patients (73%) had a non-liver-related comorbidity at the baseline, and the majority of these patients suffered from hypertension and/or diabetes. Overall at the time of receipt of ^90^Y radioembolization, there were 6 patients who were receiving Sorafenib concurrently, and an additional 11 patients had received Sorafenib prior to ^90^Y therapy. With the exception of Sorafenib, no other concurrent locoregional or systemic HCC-directed therapy was received.

**Table 1:** Patient baseline demographics and clinical characteristics at enrollment (*n* = 30)

Patient Characteristics		Parameters	Overall n (%)	1 month PCS within 2SD of NBS (*n* = 18)	1 month PCS > 2SD below NBS (*n* = 12)	*p*-value
Age		Median (range)	62 (35–82)	60.8	61.3	0.46
Gender		Male	23 (77%)	13	10	0.31
		Female	7 (23%)	5	2	
Ethnicity		White	24 (80%)	14	10	0.37
		Black	4 (13%)	3	1	
		Other	2 (7%)	1	1	
HCC Etiology		HCV	16 (53%)	10	6	0.42
		HBV	2 (7%)	1	1	
		ALD	5 (17%)	3	2	
		Other causes of cirrhosis	7 (23%)	4	3	
Non-Liver Comorbidities (e.g. Hypertension, Diabetes)		Present	22 (73%)	14	8	0.25
		Absent	8 (27%)	4	4	
Portal Hypertension		Present	20 (67%)	11	9	0.43
		Absent	10 (33%)	7	3	
Hepatic Encephalopathy		Present	3 (10%)	1	2	0.33
		Absent	27 (90%)	17	10	
Child-Pugh Class		A	20 (67%)	11	9	0.19
		B	10 (33%)	7	3	
		C	0 (0%)	0	0	
ECOG Performance Status		0	13 (43%)	9	4	0.17
		1	17 (57%)	9	8	
Tumor Morphology	Tumor Locations	Unilobar	19 (63%)	12	7	0.82
		Bilobar	11 (37%)	6	5	
	Number of Nodules	Solitary	11 (37%)	7	4	0.23
		Multiple	19 (63%)	11	8	
	Mean largest tumor size (cm)		**9.2 (4.9–19)**	**9.7**	**9.1**	**0.04**
	Mean largest tumor volume (cm^3^)		588 (145–1136)	580	602	0.42
	Tumor Burden	<50%	20 (67%)	11	9	0.20
		50%–75%	10 (33%)	7	3	
		>75%	0 (0%)	0	0	
Portal Vein Thrombosis (PVT)	Presence	Present	30 (100%)	18	12	0.26
		Absent	0 (0%)	0	0	
	Location	Main PV	6 (20%)	4	2	0.63
		Branch PV	24 (80%)	14	10	
	Degree of Occlusion	Occlusive	23 (77%)	13	10	0.30
		Non-Occlusive	7 (23%)	5	2	
Laboratory Data	Mean Serum AFP (ng/dl)		715 (3.3 to >2400)	703	801	0.36
Previous Treatment		No	10 (33%)	7	3	0.22
		Yes	20 (67%)	11	9	
Type of Previous Treatment^a^		cTACE/DEB-TACE	14 (47%)	10	4	0.19
		RFA	0 (0%)	0	0	
		Liver Resection	0 (0%)	0	0	
		Sorafenib^a^	6 (20%)	4	2	
Lung Shunt Fraction (LSF)		Mean (%)	11.0 (5.1–19.8)	11.3	10.8	0.52

*PCS* Physical Component Summary Scores, *SD* Standard Deviation, *ECOG* Eastern Cooperative Oncology Group, *PVT* Portal Vein Thrombosis, *cTACE* Conventional Transarterial Chemoembolization, *DEB-TACE* Drug-Eluting Bead Transarterial Chemoembolization, *RFA* Radiofrequency Ablation

^a^Indicates number of patients who were taking Sorafenib at the time of ^90^Y. A total of 17 patients (55%) had been on Sorafenib at the time of or prior to receiving ^90^Y therapy

Bold data are statistically significant (*P* <0.05)

### SF-36 survey results

**Fig. 1:**
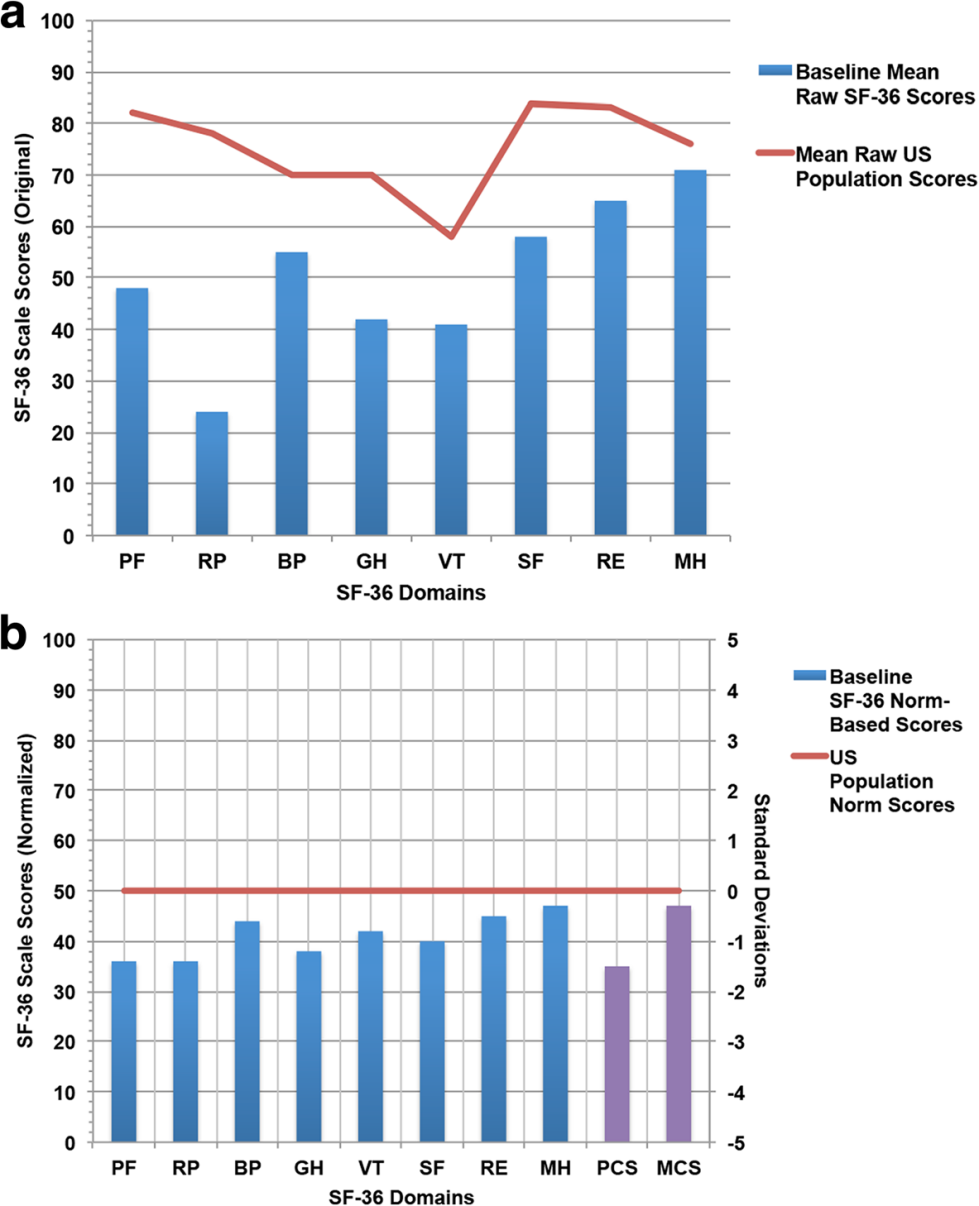
Comparison of mean SF-36 HRQOL Normalized Baseline Scores for patients with HCC prior to Y90 therapy (bars) versus mean SF-36 HRQOL scores of an age-adjusted healthy US population (lines). **a** Raw SF-36 scores compared to US population means; (**b**) Normalized SF-36 scores. Significantly lower HRQOL scores were observed in patients at baseline compared to the mean US healthy population (*p* < 0.001). PF: Physical Functioning; RP: Role-Physical; BP: Bodily Pain; GH: General Health; VT: Vitality; SF: Social Functioning; RE: Role Emotional; MH: Mental Health; MCS: Mental Component Summary Score; PCS: Physical Component Summary Score

Of the 30 patients, all patients completed 1-month follow-up surveys, with 26 (87%) and 20 (67%) having completed 3-month and 6-month follow-up surveys respectively at time of data collection. The decline in response rate at was due to 4 patient deaths at 3 months and 10 patients deaths by 6 months. Twenty-three patients (77%) were deceased at the time of data analysis. The median time interval after initial ^90^Y radioembolization therapy to the first follow-up survey was 4.5 weeks. Median follow-up time for all patients was 17.4 months following initial ^90^Y therapy. Lower baseline raw SF-36 scores in each of the 8 domains were observed on comparison to the raw scores of a healthy, age-adjusted healthy population in the US (Fig. 1a). Additionally, the mean SF-36 HRQOL Normalized Baseline Scores (NBS) in all domains were significantly lower when compared to mean scores of the healthy, age-adjusted population in the US (*p* < 0.001) (Fig. 1b). Baseline physical component scores, including Physical Functioning (PF) and Role-Physical (RP), as well as the overall Physical Component Summary Score (PCS) were more substantially decreased vs. age-adjusted norms compared to mental component scores (Fig. 1b).

### HRQOL analysis

**Fig. 2:**
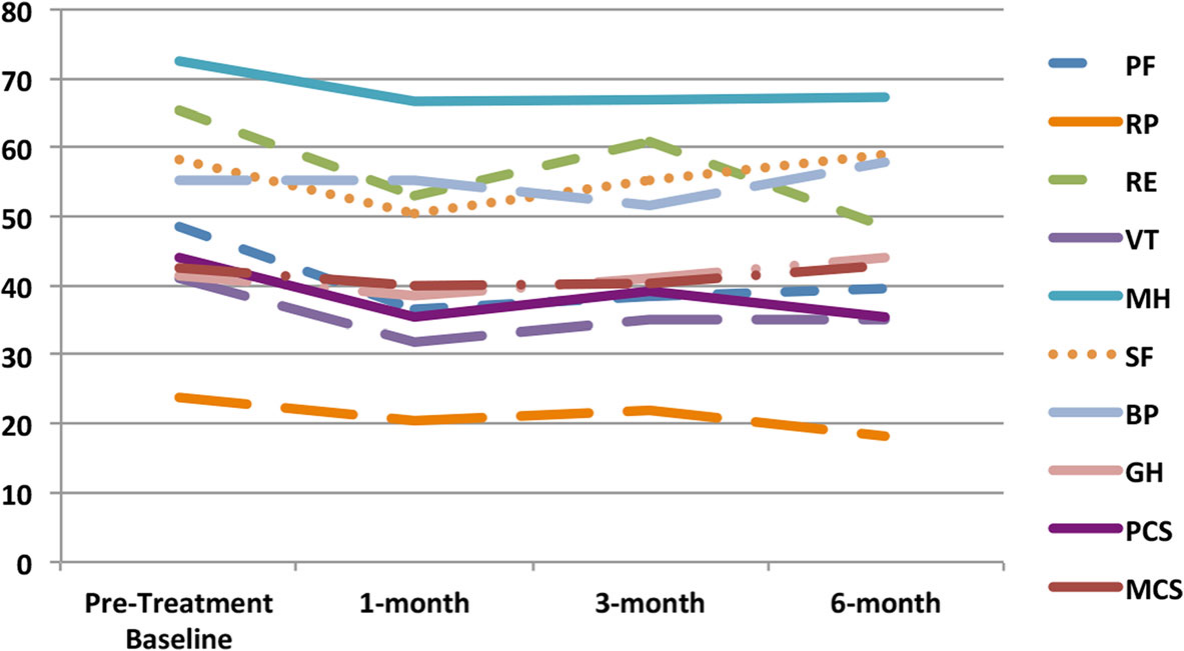
Longitudinal analysis of HRQOL after Y90 radioembolization demonstrating lack of significant change in health-related quality of life pre- and post-Y90 therapy, and over the course of 6-month follow-up. PF: Physical Functioning; RP: Role-Physical; BP: Bodily Pain; GH: General Health; VT: Vitality; SF: Social Functioning; RE: Role Emotional; MH: Mental Health

There were no significant changes in any of the 8 domains at 1-month, 3-month and 6-months post-^90^Y therapy compared to pre-^90^Y baseline scores on longitudinal analysis (Fig. 2). When comparing scores at 1-month vs. 3 months post-^90^Y therapy, there were non-statistically significant positive trends in Role-Physical (20.37 vs. 22.06, *p* = 0.27) and Vitality (31.85 vs. 35.22, *p* = 0.11) domains; however, a significant increase in the Role-Emotional score (53.09 vs. 60.78, *p* = 0.04) was observed. At 3 months post-^90^Y therapy, a negative trend in the General Health domain (44.09 vs. 41.52, *p* = 0.07) almost reached significance. Longitudinal analysis of all 8 domains (Fig. 2) demonstrated no significant changes between follow-up in the short-term within 1–3 months vs. 6-month scores. A comparison of 3-month vs. 6-month follow-up scores revealed a nonsignificant decrease in the Role-Emotional domain (60.78 vs. 48.48, *p* = 0.06) and an increase in the Bodily Pain domain (51.62 vs. 57.95, *p* = 0.08). No significant changes in either the physical component summary scores (PCS) or mental component summary scores (MCS) were observed at all follow-up intervals when compared to baseline, or between 1- vs. 3-month or 3- vs. 6-month interval scores (*p* > 0.05 for all).

### ^90^Y Radioembolization and physical component summary scores

Baseline characteristics were further stratified based on PCS at 1-month post-^90^Y therapy (Table 1). Eighteen patients (60%) had 1-month PCS within 2 standard deviations (SD) of US age-adjusted healthy normalized baseline scores, while 12 patients (40%) had 1-month PCS greater than 2 SD below that of US age-adjusted healthy NBS. No significant differences in baseline characteristics were found between patients who had 1-month post-^90^Y PCS within 2SD vs. >2SD below that of mean NBS. However, it was noted that baseline mean largest tumor size was significantly greater in patients with 1-month PCS within 2SD of NBS (9.7 cm vs. 9.1 cm, *p* = 0.04).

### Survival and outcomes

**Fig. 3:**
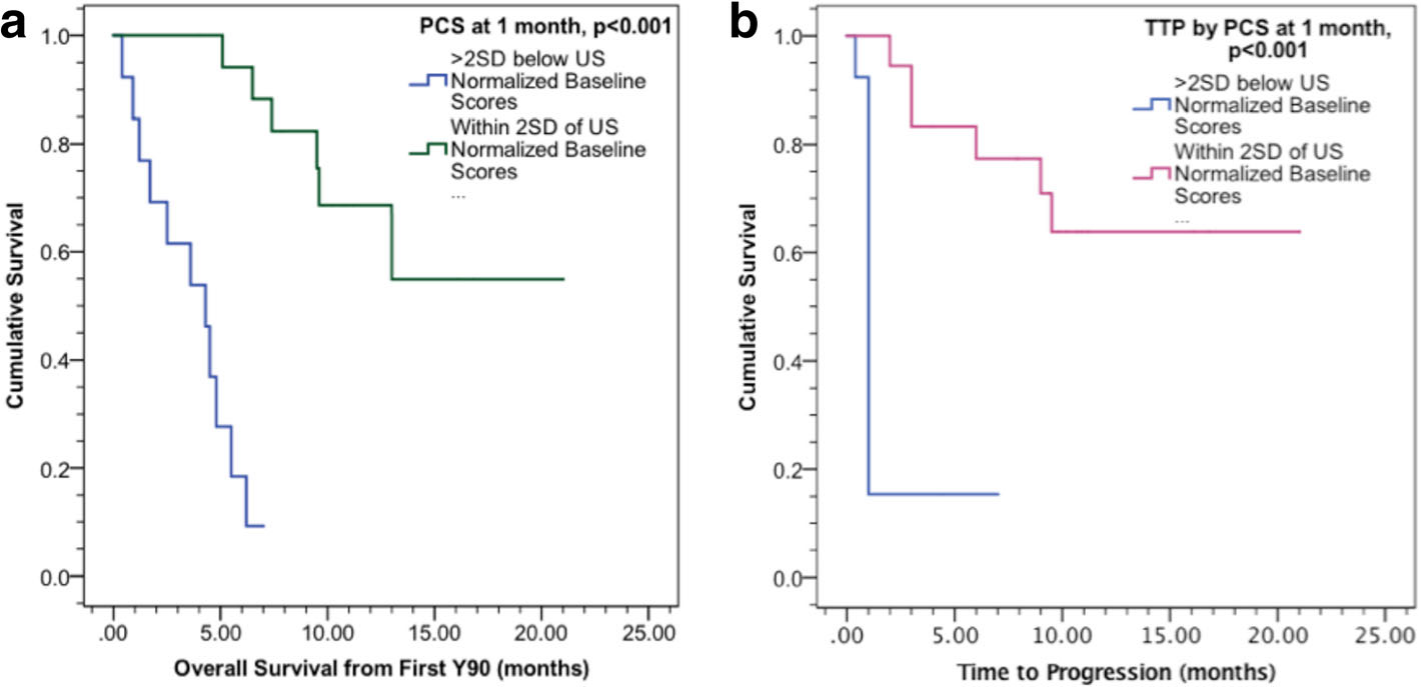
Kaplan Meier analysis of HRQOL for patients who had PCS within 2 standard deviations (SD) vs. those with PCS >2SD below US normalized baseline scores. At 1 month post-Y90, patients with PCS demonstrated: (**a**) Significantly prolonged median OS (15.7 months vs. 3.7 months; *p* < 0.001); and (**b**) Significantly prolonged time to progression (12.4 months vs. 1.8 months; *p* < 0.001) compared those with physical component scores >2SD below normalized US population values

The median OS from initial ^90^Y radioembolization was 13.0 months (95% confidence interval (CI), 4.4–22 months) while median overall time to progression was 9.0 months (95% CI 6.2–13.1 months). Patients with PCS within 2SD of NBS demonstrated significantly prolonged median OS (15.7 months vs. 3.7 months, *p* < 0.001; Fig. 3a) and significantly prolonged TTP (12.4 months vs. 1.8 months, *p* < 0.001; Fig. 3b) when compared those with PCS >2SD below the healthy, normalized US population.. Overall 3-month and 6-month survival rates from ^90^Y therapy were 86.7% and 66.7%, respectively. Three-month survival rates for patients with 1 month post-^90^Y PCS within 2SD was 94.4%, compared to 83.3% for patients with 1 month post-^90^Y PCS >2SD below NBS, *p* = 0.01. Six-month survival rates were 72.2% vs. 58.3% for patients with 1 month post-^90^Y PCS within 2SD vs. >2SD below NBS, *p* = 0.005. On multivariate analysis, ECOG performance score (hazard ratio (HR) 11.29, *p* = 0.021), Child Pugh class (HR 6.32, *p* = 0.013), Lung Shunt fraction (HR 6.42, *p* = 0.029), and PCS at 1-month post-^90^Y therapy (HR 1.73, *p* = 0.041) were significant independent predictors of overall survival. In addition, these same factors were also significant independent prognostic factors for time to progression (Table 2).

**Table 2:** Univariate and Multivariate Analysis of Prognostic Factors for Overall Survival and Time-to-Progression of Disease

Prognostic Factor	Parameters Compared	Overall Survival (OS)				Time to Progression (TTP)			
		Univariate Analysis		Multivariate Analysis		Univariate Analysis		Multivariate Analysis	
		HR (95% CI)	*p*-value	HR (95% CI)	*p*-value	HR (95% CI)	*p*-value	HR (95% CI)	*p*-value
Age	>65 vs. ≤65 years	1.13 (0.44–2.94)	0.12	N/A	–	1.14 (0.44–2.95)	0.80	N/A	–
Gender	Male vs. Female	1.28 (0.41–3.98)	0.67	N/A	–	1.19 (0.39–3.66)	0.76	N/A	–
Comorbidity	Present vs. Absent	0.80 (0.26–2.45)	0.69	N/A	–	0.98 (0.32–3.00)	0.97	N/A	–
Portal Vein Thrombosis	Main vs. Branch Portal Vein	1.70 (0.55–5.25)	0.36	N/A	–	1.68 (0.55–5.17)	0.37	N/A	–
	Occlusive vs. non-occlusive	1.23 (0.39–3.82)	0.72	N/A	–	1.19 (0.39–3.66)	0.76	N/A	–
Tumor Size	>10 vs. ≤10 cm	1.02 (0.39–2.65)	0.97	N/A	–	0.89 (0.34–2.31)	0.81	N/A	–
Tumor Volume	>500 vs. ≤500 cm^3^	1.34 (0.48–3.43)	0.87	N/A	–	1.12 (0.73–3.15)	0.72	N/A	–
Tumor Extent	Unilobar vs. Bilobar	1.02 (0.38–2.77)	0.96	N/A	–	1.08 (0.40–2.93)	0.88	N/A	–
	Solitary vs. Multifocal	1.43 (0.61–4.15)	0.76	N/A	–	1.12 (0.43–2.17)	0.69	N/A	–
Portal Hypertension	Present vs. Absent	2.69 (0.77–9.40)	0.12	N/A	–	2.53 (0.73–8.80)	0.146	N/A	–
Encephalopathy	Present vs. Absent	1.45 (0.33–6.44)	0.62	N/A	–	1.38 (0.31–6.05)	1.38	N/A	–
ECOG PS	0 vs. 1–2	**16.17 (2.06–126.83)**	**0.018**	**11.29 (1.92–19.06)**	**0.021**	**13.01 (1.69–100.4)**	**0.016**	**13.52 (1.65–175.27)**	**0.004**
Child Pugh Class	A vs. B	**8.21 (2.59–26.04)**	**0.006**	**6.32 (1.79–16.55)**	**0.013**	**7.07 (2.17–23.00)**	**0.009**	**12.31 (2.58–59.23)**	**0.005**
Alpha-fetoprotein	>400 vs. ≤400 ng/dl	1.12 (0.43–2.97)	0.82	N/A	–	1.06 (0.40–2.78)	0.911	N/A	–
Lung Shunt Fraction	>10% vs. ≤10%	**4.89 (1.55–15.63)**	**0.004**	**6.42 (1.96–10.31)**	**0.029**	**3.91 (1.33–11.36)**	**0.013**	**7.91 (3.06–17.48)**	**0.011**
Treatments prior to Y-90	Yes vs. No	1.67 (0.37–3.18)	0.35	N/A	–	1.74 (0.51–2.93)	0.41	N/A	–
Baseline MCS	Within 2SD vs. >2SD below of NBS	1.25 (0.22–2.57)	0.38	N/A	–	1.18 (0.77–3.68)	0.36	N/A	–
Baseline PCS	Within 2SD vs. >2SD below of NBS	1.38 (0.66–3.39)	0.58	N/A	–	1.29 (0.93–3.17)	0.19	N/A	–
MCS at 1 month post-Y90	Within 2SD vs. >2SD below of NBS	2.24 (0.98–3.98)	0.17	N/A	–	2.13 (0.53–3.66)	0.83	N/A	–
PCS at 1 month post-Y90	Within 2SD vs. >2SD below of NBS	**2.59 (1.04–4.23)**	**0.026**	**1.73 (1.02–3.18)**	**0.041**	**2.03 (1.13–4.57)**	**0.023**	**3.28 (1.48–5.13)**	**0.044**
MCS at 3-months post-Y90	Within 2SD vs. >2SD below of NBS	1.87 (0.49–2.13)	0.83	N/A	–	1.55 (0.90–2.98)	0.88	N/A	–
PCS at 3 months post-Y90	Within 2SD vs. >2SD below of NBS	1.48 (0.86–3.02)	0.44	N/A	–	1.47 (0.74–2.93)	0.59	N/A	–
MCS at 6 months post-Y90	Within 2SD vs. >2SD below of NBS	1.44 (0.82–2.73)	0.23	N/A	–	1.96 (0.42–4.13)	0.38	N/A	–
PCS at 6-months post-Y90	Within 2SD vs. >2SD below of NBS	1.59 (0.92–2.11)	0.71	N/A	–	1.37 (0.79–3.13)	0.62	N/A	–

Bold data are statistically significant (*P* <0.05)

## Discussion

This study examined the efficacy of ^90^Y radioembolization therapy on HRQOL in patients with advanced, infiltrative HCC with PVT. Currently, ^90^Y radioembolization is considered a viable therapeutic option for palliative treatment of advanced stage HCC patients who are not candidates for surgery. As our results demonstrated, such patients tended to have baseline HRQOL scores significantly below that of the normal age-adjusted healthy population. Given the overwhelmingly poor prognosis of infiltrative HCC with PVT and the tendency to have exhausted other therapeutic options in such patients, it is imperative to ensure that any treatment rendered does not further worsen patients’ quality of life.

The survival benefits of ^90^Y radioembolization therapy on HRQOL in infiltrative, advanced HCC with PVT have not been extensively characterized in the literature for adequate comparison with the current study. However, in a cohort of patients with majority intermediate-advanced (BCLC B/C) disease, ^90^Y radioembolization therapy gave rise to significant increases in social and functional well-being at 1 month, without significant changes in overall HRQOL scores [21]. In addition, for HCC patients without PVT, ^90^Y radioembolization therapy improved HRQOL scores compared to cisplatin administered through hepatic arterial infusion (HAI) [24], with significantly higher functional well being at 3-months and 6-months post-^90^Y therapy vs. cisplatin HAI. Previous studies investigating HRQOL patients receiving other forms of intra-arterial therapy such as transarterial embolization or cTACE have reported decreased HRQOL was at 3 month follow-up compared to baseline [25, 26], and improved HRQOL at 6 months vs. 3 months [25]. A study by Wible et al. [20] indicated that HCC patients who received ≥3 cTACE procedures had significantly improved mental health scores following the initial procedure (13 point change, *n* = 21; *p* = 0.008) as well as the second procedure (12.2 point change, *n* = 23; *p* = 0.002). In addition, significant improvements in bodily pain (9.9 point change, n = 21; *p* = 0.047) after the first procedure were observed.

HRQOL is increasingly being used as an important prognostic tool in palliative oncology, and some have reported that it may be just as useful as objective outcomes such as survival or tumor response [2, 27, 28]. When examining patients with chronic liver disease in the presence or absence of HCC, Kondo et al. found that even though HCC patients had worse physical and overall HRQOL, it was liver function and not the presence of HCC that served as main determinant of HRQOL [24, 29, 30]. Our study demonstrated positive trends in HRQOL, particularly in the Role-Emotional, Role-Physical, Vitality and Social Functioning domains. Concurrently, there were no significant decreases in any of the remaining domains at 1–3-, or 6-months following treatment. This suggests that it is possible to treat with advanced, infiltrative HCC with PVT with ^90^Y radioembolization and increase survival without significant concern about further decreases in HRQOL in these patients.

Our study also demonstrated a median OS of 13.0 months from initial ^90^Y radioembolization and a median time to progression of 9.0 months. The survival outcomes of ^90^Y radioembolization for infiltrative HCC with PVT have not been widely reported. However, given that all patients in this study were BCLC Stage C, such survival times are comparable with other published studies assessing the efficacy of DEB-TACE in HCC, with median OS of 13.3 months [31] to 13.5 months [32] in BCLC Stage C patients alone. Prior studies of non-infiltrative HCC patients with PVT reported median OS of 10 to 13 months [33, 34]. In our study, patients with PCS within 2SD of NBS demonstrated significantly increased median OS (15.7 months vs. 3.7 months, *p* < 0.001) and significantly prolonged time to progression (12.4 months vs. 1.8 months, *p* < 0.001) compared those with PCS >2SD below a healthy, age-adjusted US population. This was the case despite the fact that patients with PCS within 2SD of NBS had significantly higher mean index tumor size (9.7 cm vs. 9.1 cm, *p* = 0.04) compared to patients with PCS >2SD below normalized US population. Furthermore, PCS at 1-month post-^90^Y radioembolization was a significant independent prognostic indicator for prolonged overall survival (HR 1.73; 95% CI 1.02–3.18; *p* = 0.041) and time to progression (HR 3.28; 95% CI 1.48–5.13; *p* = 0.044), and significant decreases in PCS were independent predictors of poorer OS and TTP. In addition to the highlighting the value of physical component assessments in predicting patient survival, these results indicate the potentially integral role HRQOL analysis may play in comparative effectiveness analysis of therapeutic options.

The strengths of this study included its prospective nature as a correlative study with a phase II randomized controlled trial, with extensive short- and medium-term follow-up of patient outcomes in advanced, infiltrative HCC with PVT. Some limitations included the study’s small sample size, the fact that not all patients were able to complete surveys at all follow-up intervals, due either to worsening clinical status or death and subsequent loss to follow-up. In addition, as all HRQOL survey measures are dependent on multifactorial internal and external influences, possible bias (including reporter and observer bias) and confounding factors cannot be completely ruled out.

## Conclusions

In summary, our study demonstrated that health-related quality of life was preserved in the short- and medium-term for patients who received ^90^Y radioembolization for infiltrative, advanced HCC with PVT. Furthermore, ECOG performance status, Child-Pugh score, lung shunt fraction and 1-month post-^90^Y therapy physical component summary scores were independent prognostic factors for overall survival from therapy.

## Abbreviations

BCLC: Barcelona Clinic Liver Cancer
CLIP: Cancer of the Italian Liver Programme
cTACE: Conventional transarterial chemoembolization
DEB-TACE: Drug eluting bead transarterial chemoembolization
HCC: Hepatocellular Carcinoma
HRQOL: Health-related quality of life
MELD: Model for End-Stage Liver Disease
PVT: Portal vein thrombosis
SF-36: Short-Form 36
UNOS: United Network for Organ Sharing
Y90: Yttrium-90 Radioembolization
